# REPORTED HISTORY OF ABORTION AS A CONTRIBUTOR OF RELIGIOSITY AMONG OLDER WOMEN HELD IN CORRECTIONAL CUSTODY

**DOI:** 10.1093/geroni/igad104.3084

**Published:** 2023-12-21

**Authors:** Hailey Minet, Brianna Barnett, Alexander Bishop, Kevin Randall

**Affiliations:** Oklahoma State University, Stillwater, Oklahoma, United States; Oklahoma State University, Stillwater, Oklahoma, United States; Oklahoma State University, Stillwater, Oklahoma, United States; Sam Houston State University, Huntsville, Texas, United States

## Abstract

This study assessed the interconnection between abortion, forgiveness, and social provisions on religiosity for N = 157 older women (45 years and older) under correctional custody in Oklahoma. Guided by the Model of Developmental Adaptation, path analysis was conducted positing positive religiosity as the developmental outcome, regressed on proximal influences assessed by social provisions and forgiveness and the distal influence, the independent predictor, whether or not a woman had ever had an elective abortion in their lifetime. Endogenous measures were controlled for age, race, marital status, education, and crime type. Using Mplus 8.8 and an estimator that provides parameter estimates and a chi-square test statistic robust to non-normality, the model fit well with a non-significant chi square test statistics and significant parameter estimates as expected. Reported history of abortion was significantly associated with forgiveness (β= .25, p < .05) and social provisions (β = .31, p < .05). Forgiveness was also significantly associated with religiosity (β = .32, p < .01). Mediating paths were not significant. Among these women, 37% of the developmental outcome, religiosity was explained primarily by forgiveness. It appears that older women in correctional custody who have received an elective abortion have a greater disposition to engage in forgiveness and seek social support. A greater disposition to forgive also directly contributes to increased religious activity among these same women. Results have implications relative to how forensic counselors, correctional social workers, and prison chaplains provide intervening services and rehabilitation programming to sustain quality-of-life for older women aging-in-custody.

